# Sexual and social stimuli elicit rapid and contrasting genomic responses

**DOI:** 10.1098/rspb.2007.1454

**Published:** 2007-12-05

**Authors:** Molly E Cummings, Jonah Larkins-Ford, Christian R.L Reilly, Ryan Y Wong, Mary Ramsey, Hans A Hofmann

**Affiliations:** 1 Section of Integrative Biology, University of TexasAustin, TX 78712, USA; 2 Department of Biological Sciences, Hopkins Marine Station, Stanford UniversityPacific Grove, CA 93950, USA; 3 Institute for Cellular and Molecular Biology, University of TexasAustin, TX 78712, USA

**Keywords:** mate choice, behavioural genomics, female preference, neural response

## Abstract

Sensory physiology has been shown to influence female mate choice, yet little is known about the mechanisms within the brain that regulate this critical behaviour. Here we examine preference behaviour of 58 female swordtails, *Xiphophorus nigrensis,* in four different social environments (attractive and unattractive males, females only, non-attractive males only and asocial conditions) followed by neural gene expression profiling. We used a brain-specific cDNA microarray to identify patterns of genomic response and candidate genes, followed by quantitative PCR (qPCR) examination of gene expression with variation in behaviour. Our microarray results revealed patterns of genomic response differing more between classes of social stimuli than between presence versus absence of stimuli. We identified suites of genes showing diametrically opposed patterns of expression: genes that are turned ‘on’ while females interact with attractive males are turned ‘off’ when interacting with other females, and vice versa. Our qPCR results identified significant predictive relationships between five candidate genes and specific mate choice behaviours (preference and receptivity) across females exposed to males, with no significant patterns identified in female or asocial conditions or with overall locomotor activity. The identification of stimulus- and behaviour-specific responses opens an exciting window into the molecular pathways associated with social behaviour and mechanisms that underlie sexual selection.

## 1. Introduction

Across many taxa, females discriminate among suitors to select a mate and it is variation in this behaviour that facilitates both within and between species evolution. For decades, much behavioural research has been devoted to identifying the specific male phenotypes or signalling traits that stimulate the sexual interest of females (reviewed in Andersson (1994)). However, more recently researchers have begun to identify the physiological and neural processes underlying female choice. For instance, we now know how sensory systems can influence mate choice (Ryan *et al.* 1990*a*) and the use of neural activity markers has identified brain regions that process acoustic mating signals (Gentner *et al.* 2000; Sockman *et al.* 2002; Hoke *et al.* 2005). However, the genes involved in regulating mate choice behaviour in the vertebrate brain are yet to be determined. Here, we combine the robust mate choice behavioural paradigm in the swordtail, *Xiphophorus nigrensis*, a classic system in sexual selection studies, with monitoring genome scale transcriptional changes in the brain to identify the molecular components involved in mate preference behaviour. With this approach, we begin to identify how real-time molecular responses vary within the brain with variation in preference behaviour—providing the foundation for identifying biases in the central nervous system that may offer insight into the evolution of mate choice.

Poeciliids, the freshwater teleost family to which swordtails belong, are an excellent system to examine mate choice. For one, relatively simple behavioural assays in the laboratory are excellent predictors of mating success (Houde 1988) and successfully predict the changes in male genotypic frequencies observed in the wild (Endler 1983; Ryan *et al.* 1990*b*). The swordtail species in this study, *X. nigrensis*, has males of three size classes that differ in phenotype, mating behaviour and attractiveness to females. Large- and intermediate-sized males have ornaments (swords and conspicuous coloration such as UV), court females and are differentially preferred by females over the small male class that lacks ornamentation and relies on force copulations to acquire mates (Ryan & Rosenthal 2001). In preference trials in the laboratory, females exhibit behaviours in these non-contact trials that they also exhibit preceding copulation events in more naturalistic settings (Cummings & Mollaghan 2006), making behavioural assays of preference and receptivity easy to quantify without requiring physical interactions between males and females (figure 1). The ability to assess preference without contact is important to isolate the neural and molecular processes associated with choice, independent of physiological feedback associated with mating or contact with males (e.g. increase in immune response in *Drosophila* as observed by Lawniczak & Begun (2004) and McGraw *et al.* (2004)).

In this study, we combine the robust laboratory measures of preference with neural transcriptional profiles to identify possible candidate genes involved in regulating this behaviour. Further, we examine the association between differences in mate preference behaviour and quantitative variation in levels of candidate gene expression in individual females. In a non-contact arena, allowing visual assessment only, we recorded behavioural responses of females exposed to a pair of males differing in attractiveness (large versus small males, LS), two females (female control, FF), two unattractive males (small male control, SS) and two empty compartments (asocial control, AA) followed by whole brain gene expression profiling to a brain-specific cDNA microarray (experiment 1) and real-time quantitative PCR (qPCR; experiment 2). Experiment 1 identified genome-scale responses as well as possible candidate genes for specific social conditions (e.g. mate preference behaviour towards attractive males), and experiment 2 examined the relationship of individual-level variation in behaviour (mate choice-specific and general locomotor activity) to variation in levels of candidate gene expression.

## 2. Material and methods

### (a) Behavioural experiments

All behavioural trials were conducted in a dichotomous choice setup where stimuli (one out of the four types, see below) are placed behind UV transparent plexiglas in the end compartments of an experimental aquarium that mimics the optical environment of their natural habitat in the Rio Choy, San Potosi, Mexico (as in Cummings *et al.* (2003); figure 1). Each of the 58 focal females (28 from experiment 1 and 30 from experiment 2) were placed in the middle compartment which was divided into three observational zones: 24 cm areas near each end compartment (association zones) and a 24 cm ‘neutral’ zone in the centre. After a 5-min female acclimation period in the neutral zone, we recorded female association time (time spent in each association zone) and behaviour (e.g. glide response) for 15 min. Glide responses, a slow swim away coupled with a quick return to face the male that often precedes a copulation event in free-ranging environments (Cummings & Mollaghan 2006), were used as a measure for receptivity displays and are similar in function to receptivity responses in other poeciliids (e.g. Houde 1997). After 15 min, stimuli are then switched (to control for side biases) and behaviour is recorded for another 15 min. Following behavioural trials, we evaluated association bias (proportion of association time spent with individual *a*, where time with *a* is greater than time with *b*) as well as a preference score that incorporated both association bias and glide responses (see experiment 2). Immediately following each trial (30 min of exposure to stimuli), focal females were sacrificed via decapitation following IACUC protocol no. 04100402 and whole brains were dissected and stored in RNAlater (Ambion) for RNA extraction.

To identify the genomic factors underlying female choice responses, we compared behavioural and genomic response of females across four social stimuli: (LS) pairing an attractive (large male 41.5, 42 or 38 mm in standard length (SL)) with an unattractive male (small male 24, 24 or 21 mm), respectively, to simulate a female choice environment with a salient preference; (SS) a male exposure condition where attraction to stimuli, and presumably preference behaviour, was minimal (pairing two small males: 20 and 20 mm in SL); (FF) a female exposure condition to account for socialization with conspecifics in a non-mate preference environment (pairing two females: 22 and 22.5 mm in SL); and (AA) an asocial condition to account for experimental conditions independent of social interactions (empty stimuli compartments). The SS condition served three purposes: (i) to provide a male exposure group in which preference behaviour is unlikely, thereby accounting for female reaction to male exposure independent of preference; (ii) to provide a male exposure group in which the stimuli size is the roughly same as the FF group—thus removing any possible size confound from sex-specific genomic responses; and (iii) to provide a continuum of response for preference behaviour towards males from minimal (SS) to high (LS) allowing for regression analysis.

We conducted two different experiments using these four behavioural stimuli. In experiment 1, we used microarray hybridization to identify candidate genes associated with exposure to attractive males relative to other social interactions. Since microarray hybridizations compare the relative expression of two groups, we used a reference design where we competitively hybridized each of the three social controls (SS, FF and AA) to LS treatment. We then sequenced and cloned some of the candidate genes associated with the LS response to further explore the relationship between gene expression and behaviour. In experiment 2, we used qPCR to examine how expression for these genes varied with changes in behaviour (preference, receptivity or locomotor activity).

### (b) Experiment 1: using microarray to identify candidate genes

We isolated 28 adult female *X. nigrensis* from males for two to six weeks to ensure reproductive receptivity, prior to assigning each to one out of the four stimuli groups (seven females per LS, SS, FF and AA). Given that females can exhibit a range of preference responses (e.g. Cummings & Mollaghan 2006), we selected seven females for the LS group that represented a range of responses (from low (less than 0.65), intermediate (0.70–0.85) and high (more than 0.85) association bias) rather than selecting at random or biasing this group with females of only high preference. Our rationale for selecting for a continuum rather than at random was for subsequent within-group comparison of individual level variation in preference behaviour and genomic response; however, we were unable to perform these analyses in the microarray experiment (experiment 1) due to the necessity of pooling RNA within groups. Owing to time constraints, we pre-tested only 15 out of the 28 females to identify females of high and low association bias for large versus small males. Each female was exposed to the same pair of males on three separate trial days, and we selected the seven females for the LS group according to their mean association bias (0.54, 0.61, 0.62, 0.76, 0.82, 0.87 and 0.88). The other eight pre-tested females showed a similar range in association bias responses as the seven LS selected females (Mann–Whitney *U*=16, *p*=0.165), and were randomly assigned to the three social control groups (SS, FF and AA) along with the remaining 13 isolated females.

### (c) Experiment 1: microarray hybridizations

We used a cDNA microarray platform (NCBI GEO platform GPL928) constructed with randomly selected clones from a brain-derived cDNA library obtained from the African cichlid fish *Astatotilapia burtoni.* This platform has previously been shown to give biologically meaningful results in heterologous hybridizations using poeciliid RNA (Renn *et al.* 2004). To evaluate the performance of a cichlid array platform with our *X. nigrensis* experimental tissue, genomic DNA hybridizations were performed to compare hybridization quality between the platform and *X. nigrensis*. Genomic DNA (gDNA) was extracted from *X. nigrensis* (Xn) and *A. burtoni* (Ab, microarray platform species), fluorescently labelled and self-hybridized (Xn versus Xn, Ab versus Ab) to the microarray according to a standard protocol (Renn *et al.* 2004). Arrays were normalized in R/Bioconductor with print-tip loess after background correction (within arrays) and scanned with an Axon 4000B (Axon Instruments) scanner. Resulting images were processed in GenePix v. 5.0 (Axon Instruments).

Despite 65 Myr since cichlids and poeciliids shared a common ancestor, 4227 out of 4573 array features (92.4%) gave hybridization signals that were 2 s.d. above background, suggesting a very high degree of heterologous hybridization with this platform. Comparison of log2 intensity ratios from self-hybridizations of either *A. burtoni* or *X. nigrensis* allowed us to identify ‘noisy’ spots (probably due to sequence divergence). There was significantly less variation in the *A. burtoni* labelled gDNA hybridizations compared with *X. nigrensis* (*F*-test: *F*=406.91, *p*≤0.0001). We excluded all spots from the *X. nigrensis* expression analysis, whose gDNA ratios fell outside±2 s.d. of the *A. burtoni* distribution (*n*=554 or 13% of those that were above background).

After the gDNA comparisons between species, we prepared our experimental tissue for cDNA microarray hybridizations. After homogenizing the experimental tissue, RNA was extracted using Trizol (Invitrogen). Total RNA from each individual brain was insufficient for between individual hybridizations, and sample pooling was required along with a reference design to minimize the total number of competitive hybridizations. Since our objective was to identify genomic response during mate choice-like conditions relative to other social interactions, we pooled all seven brains from the LS condition and used them as the reference in the microarray experiment. Using the LS condition as our reference enabled us to directly compare genomic responses in asocial, female only and unattractive male conditions with genomic responses with an attractive male present. For the other three groups (SS, FF and AA), 200 ng of total RNA per individual was combined from three or four individuals, creating two biological replicates per condition. The RNA from each replicate was linearly amplified with one round of *in vitro* transcription using an ExpressArt mRNA amplification kit (Artus Biotech) according to the manufacturer's instructions. For each competitive hybridization event, 140 ng of amplified RNA (from the treatment pool and one of the social controls) was labelled and hybridized as in Renn *et al.* (2004). Each biological replicate was hybridized twice (with dye reversal) against the treatment reference. Hence, we used a reference design with 12 arrays and four replicates per comparison. All arrays were scanned with the Axon 4000B scanner and images processed in GenePix v. 5.0. Arrays were normalized in R/Bioconductor with print-tip loess (within arrays; Smyth & Speed 2003) and intensity ratios were calculated.

### (d) Experiment 1: microarray data analysis

After removing all sequence-diverged features, and features with average intensity less than 2 s.d. above the average background, we compressed duplicate features by calculating the averages, and used 3422 genes (90% of array) for expression analysis. We employed Bayesian Analysis of Gene Expression Levels (BAGEL, Townsend & Hartl 2002) to estimate expression differences for any given gene between the experimental groups and used a Bayesian posterior probability (PP) threshold of 0.99 or above and 0.95 or above for predictive gene analysis. BAGEL allows for the statistical estimation of relative expression levels across experimental categories (e.g. test conditions or individuals), with each category being compared to another in a direct or indirect fashion. BAGEL produces relative expression estimates and Bayesian PPs across categories normalized to the group with the lowest expression level (which is set at 1). To correct for multiple hypothesis testing, we assessed the false discovery rate (FDR) according to Benjamini & Hochberg (1995). Maybe not surprisingly, few genes (*n*=73) passed this test. This is probably due to the increased variance obtained with heterologous hybridizations. There is overall significantly more variability in the heterologous hybridizations (*F*-test: *F*=356.86, *p*<0.0001) and significant spots (exceeding Bayesian PPs, PP≥0.99) tend to be those where variability is less than average. Owing to this increased variability in the *X. nigrensis* hybridizations, we do not expect as many highly significant spots and, as a consequence, fewer genes that survive the FDR correction procedure.

In order to compare expression profiles for the different conditions and to assess the amount of biological variability within an experimental condition, we performed unsupervised hierarchical clustering with Euclidean distance as the similarity metric and complete linkage using the *hclust* function in R/Bioconductor. We clustered genes with a significant difference of PP≥0.99 across conditions and used bootstrapping to obtain confidence values for the cluster nodes. We constructed consensus trees by repeating the hierarchical clustering 1000 times on randomly permutated expression profiles using the *hclust*, *consensus* and *heatmap* functions in R/Bioconductor.

To identify candidate genes for each social condition we examined the differentially expressed genes from experiment 1 and identified genes that were significantly differentially expressed and predictive of specific social conditions (i.e. up- or downregulated in only one condition compared to all others; PP≥0.95). Out of the 77 mate choice candidate genes identified in this process, 19 are currently annotated (see table S1 in the electronic supplementary material). We selected five out of these 19 annotated genes along with *egr-1* (an immediate early gene) for further analysis either due to (i) a documented role in reproduction or social behaviour or (ii) arbitrary (e.g. the ease of cloning) basis. The five genes we cloned and sequenced from *X. nigrensis* were the *neuroserpin* precursor, *β*_*1*_*-adrenergic receptor, apyrase, neuroligin 3* and *importin*. Of note, these five genes were of only moderate range in gene expression differences relative to some of the other candidate genes not selected for further analysis (e.g. BAGEL estimated relative expression levels normalized by lowest median expression across the four groups LS : FF : SS : AA for *β*_*1*_*-adrenergic receptor* 1 : 1.8 : 1.4 : 1.6, *apyrase* 1 : 1.9 : 1.4 : 1.8; *neuroligin* 1 : 3.5 : 2.2 : 2.5, *neuroserpin* precursor 1.3 : 1 : 1.1 : 1.1, *importin* 1 : 1.1 : 1.2 : 1.1, and for annotated genes not selected for sequencing: *integrin β*_*5*_ subunit precursor protein 1 : 2.79 : 2.0 : 2.28; *nicolin 1* 1 : 2.9 : 1.96 :2.9; *β-soluble NSF attachment protein* 1 : 2.5 : 2.25 : 2.56; and *glutamine synthetase* 1 : 1.7 : 1.8 : 1.33; see table S1 in the electronic supplementary material for more details).

We then conducted qPCR assays of these five genes using nested primer strategy based on *A. burtoni* EST sequences to compliment our microarray results (http://biocomp.dfci.harvard.edu/tgi/cgi-bin/tgi/gimain.pl?gudb=a_burtoni). Primers were designed using MacVector (Accelrys) or PrimerQuest (Integrated DNA Technologies, http://www.idtdna.com/Scitools/Applications/Primerquest/). GenBank accession numbers for the six genes include *neuroserpin* precursor (DQ839542), *neuroligin 3* (DQ839541), *importin* (DQ839540), *apyrase* (DQ835283), *β*_*1*_*-adrenergic receptor* (DQ839536) and *egr-1* (DQ835282). Cloning primer sequence, clone size and homology to *A. burtoni* are shown in table S2 in the electronic supplementary material. Using mRNA remaining from experiment 1 (with remaining total RNA from four LS, three SS, five FF and six AA females) and qPCR methods described below (§2*e*), we validated the microarray relative expression patterns for four of the five sequenced genes. The relative expression of *β*_*1*_*-adrenergic receptor* across females in experiment 1 as quantified by qPCR and normalized to the lowest average group expression (LS : FF : SS : AA, 1 : 6.7 : 4.3 : 6.4) mirrors that quantified using microarray hybridizations (1 : 1.9 : 1.4 : 1.8; see above) and the difference across group mean expression is nearly significant (Kruskal–Wallis (K–W) test=7.77, *p*=0.051). Similarly *neuroligin* (1 : 5.3 : 3.2 : 5.7, K–W test=7.02, *p*=0.07), *apyrase* (1 : 2.8 : 2.3 : 4.2, K–W test=4.247, *p*=0.23) and *importin* (qPCR 1 : 10.7 : 6.2 : 9.8, K–W test=7.645, *p*=0.054) followed microarray patterns of hybridization. The qPCR expression pattern of *neuroserpin* did not mirror the microarray pattern (1 : 6.5 : 4.4 : 6.3); however, the differences in mean expression across the groups was significantly different (K–W test=8.06, *p*=0.045) using both methods.

### (e) Experiment 2: examining the relationship between genes and behaviour

We isolated 30 adult female *X. nigrensis* from males for two to four weeks to ensure reproductive receptivity, prior to assigning each to one of the four stimuli groups (seven females in SS, FF and eight females in LS, AA). For experiment 2, we again conducted pre-test for large versus small male mate choice trials and selected females that displayed the same range of bias behaviour and total association times as shown in experiment 1 LS females (more than 25-min association time, mean association bias: 0.61, 0.62, 0.64, 0.69, 0.82, 0.88, 0.88 and 1.00). The same behavioural protocol (as given in §2*a*) was applied, however, with an additional measure for overall locomotor activity within each trial. In experiment 2, the number of transits (swims) each female made through the neutral zone was recorded as an activity measure (independent of receptivity). Female preference behaviour was then evaluated relative to overall activity levels, as a composite score incorporating association time bias and intensity of receptivity displays towards preferred individuals normalized by total activity level where preference score=association time bias+log((1+glides displayed towards biased stimulus)/transits through centre)). This refined preference score accounts for variation in relative display rate and general activity across females.

To explore the relationship between candidate gene expression and behaviour we repeated the behavioural experiment with 30 female *X. nigrensis* and, using qPCR analysis of the five sequenced mate choice predictive genes, examined expression patterns of these genes concomitant with behavioural patterns for mate preference. Along with the five sequenced candidate genes, we also sequenced and cloned *egr-1* using pooled whole brain cDNA and verified it through sequencing and Blast analysis. While *egr-1* was not on the cichlid array and is therefore not considered a candidate gene for preference behaviour, it is commonly used as a neural activity marker (Worley *et al.* 1991; Burmeister *et al.* 2005) and we employed it to estimate differences in whole brain neuronal stimulation between social groups.

### (f) Real-time PCR analysis

Individual brain total RNA was extracted as for experiment 1. Prior to cDNA synthesis, individual brain total RNA was DNase treated using turbo DNA-free kit (Ambion) and single-stranded cDNA was then reverse transcribed using Superscript First-Strand Synthesis for RT-PCR kit (Invitrogen) in a 60 μl reaction. The cDNA synthesis was primed with both oligo-dT and random hexamers using a modified Invitrogen protocol for the qPCR template.

The qPCR primers were designed using MacVector (primers and parameters listed in table S2 in the electronic supplementary material). The qPCR experiments were conducted using SYBR green detection chemistry on an ABI Prism 7900 real-time PCR machine (Applied Biosystems). Each sample was run in triplicate. The qPCR run results were first analysed using the Applied Biosystems Sequence Detection System software (SDS v. 2.2.1), and gene expression levels were normalized to either total RNA input quantities (experiment 1) or cDNA input quantities (experiment 2) as measured by a RiboGreen RNA quantification assay (Hashimoto *et al.* 2004). We use this assay (e.g. Aubin-Horth *et al.* 2005) as it allows for precise determinations of RNA concentrations without housekeeping gene comparisons which have been shown repeatedly to be variable in expression across experimental conditions (Bustin 2002; Hashimoto *et al.* 2004). Relative quantification was expressed as 1/*E*^avg CT^ (critical threshold) to reflect expression/individual where *E*=PCR efficiency (*E*=10^−(1/slope)^ as in Simon (2003), and the average CT value is across three replicates. This raw value was then normalized to input quantity/individual as measured by the RiboGreen assay.

## 3. Results

In experiment 1, female total association time (time in both association zones) differed among the four experimental groups (ANOVA, *F*_3,24_=4.29, *p*=0.015) with LS and FF females showing the greater total association times (LS: mean±1 s.e.=1576±37 s; FF=1450±86 s; *p*=0.565) than either the SS (891±232 s, *p*=0.004) or AA (1092±173 s, *p*=0.034) groups. Association bias differed significantly among experimental groups (LS: mean±s.e.=0.79±0.05; FF=0.61±0.08; SS=0.64±0.07; AA=0.54±0.07; *F*_3,24_=3.06, *p*=0.047). Females exhibited the greatest bias in association time in mate choice treatment (LS) where females spent significantly more time with large than small males (mean±1 s.e. association time with large=1251±102 s; small=325±789 s; paired *t*-test: *t*_6,2_=5.18, *p*=0.002) and performed significantly more receptivity displays towards large than small males (mean±1 s.e. glides towards large=6.71±1.63; small=1.71±0.71, Wilcoxon signed-rank tests; *Z*=2.207, *p*=0.027).

Whole brain gene expression profiles of the 28 females in experiment 1 using a brain-specific microarray (Renn *et al.* 2004), and Bayesian analysis of gene expression levels (Townsend & Hartl 2002) identified 306 out of the 3422 genes on the array that showed significant differential expression at the PP>0.99 level (figure 2). Hierarchical clustering of the 306 differentially expressed genes among the four experimental conditions highlights that the greatest difference in genomic expression was between distinct classes of social stimuli (e.g. females versus attractive males) rather than presence versus absence of stimuli (e.g. attractive males versus no stimuli). Compared to the other conditions, more genes showed reduced expression than increased expression in females exposed to attractive males; the opposite pattern was produced when females were exposed to other females. When we examined these genes more closely, we found that distinct social conditions produce diametrically opposed patterns of gene regulation: genes that show decreased activity in the presence of large males are the same genes that are activated in the presence of females, and vice versa.

To identify candidate genes for specific social interactions, we selected genes that were differentially expressed in only one out of the four behavioural groups. The number of uniquely differentially expressed genes was 128, with 60% of those occurring during exposure to attractive males (LS) relative to all other social conditions (77 mate choice predictive genes; figure 3).

In experiment 2, females displayed similar social behaviours as those in experiment 1 (total association times: LS (1630±36 s), FF (1466±46 s), SS (1431±125 s) and AA (1206±100 s). The only notable difference was that as a whole, SS females in experiment 2 spent more total association time than the equivalent group of females in experiment 1 (Kruskal–Wallis test *U*=9; *p*=0.048). Also, two SS females in experiment 2 exhibited a preference for one of the small males (preference scores of 0 or above; figure 5). To isolate genomic response to socially induced stimuli and behaviour, we removed females that exhibited foraging (active searching of the substrate) or non-social behaviour (more than 10 min in neutral zone) across all three social groups: removing three females from SS (one foraging and two anti-social), two females from FF (foraging) and none from the LS group.

In experiment 2, preference scores were significantly greater in the LS group (mean preference score±1 s.e. in LS: 1.02±0.11) than either the FF (0.67±0.05, K–W test, Mann–Whitney test *U*_13_=38, *p*=0.008) or AA (0.71±0.08, K–W test *U*_16_=54, *p*=0.02) groups. However, there was no significant difference in preference scores between LS and SS (0.79±0.11, K–W test *U*_12_=22, *p*=0.31), probably due to two SS females showing a preference for one of the small males, and consequently these two groups were pooled to form a ‘male exposure group’ for further analyses.

Four out of the five of the mate choice candidate genes, along with *egr-1*, showed significant positive relationships between preference behaviour and gene expression in experimental groups where males were present independent of general locomotor activity (table 1; figures 4 and 5). All of these significant relationships remain after removing potential outliers (table 1). Removing the LS female with the highest gene expression produced statistically significant relationships for these genes (*p*<0.05, except for *apyrase* where *p*=0.07), and removing the LS female with the negative preference score actually increased the significance by a factor of 3–35 for all five genes (*neuroserpin p*=0.0001, *neuroligin 3 p*=0.00005, *importin p*=0.0002, *apyrase p*=0.0007 and *egr-1 p*=0.00007). Importantly, preference behaviour provided no explanatory power in the other control groups (FF or AA, table 1). The only gene that did not follow this pattern was the *β*_*1*_*-adrenergic receptor*. It showed no relationship to preference behaviour or physical activity. However, it was the only mate choice candidate gene that covaried with receptivity displays in the treatment group, although the relationship was not significant after a Bonferroni correction (table 1).

While preference behaviour explained much of the variation in gene expression in male exposure groups, general locomotor activity (number of transits through the centre of the experimental tank) did not correlate significantly with gene expression and explained none of the variation in ‘mate choice’ candidate genes or *egr-1* expression patterns across any of the experimental groups (figure 4; table 1). Not surprisingly, females in the asocial condition exhibited more non-social swimming activity than females in any other behavioural group (figure 4; mean±1 s.e. transits through centre: LS 27.4±9.3, SS 19.5±3.9, FF 89±16.3, AA 120±20.4; ANOVA *F*_3,21_=9.62, *p*=0.0003).

In addition to investigating the relationship between gene expression and female preference behaviour, we examined all pairwise correlations between the five mate choice candidate genes and *egr-1* expression. With the exception of the *β*_*1*_*-adrenergic receptor*, there were significant correlations between all genes that were examined (table 2).

## 4. Discussion

Examinations into the role genes play in social behaviour have identified remarkable differences in gene expression profiles among groups of individuals displaying two different behavioural phenotypes. Such studies include aggregation behaviour in juvenile fruitflies (Osborne *et al.* 1997); social affiliation in voles (Young *et al.* 1999; Lim *et al.* 2004); division of labour in honeybees (Whitfield *et al.* 2003); alternative reproductive tactics in salmon (Aubin-Horth *et al.* 2005); and social dominance in cichlids (Aubin-Horth *et al.* 2007). However, little previous work has examined how genes influence behaviour *within* phenotypes. Our study is the first to highlight the rapid and complex genomic responses of the brain associated with behaviour within a single phenotype (reproductively mature females) exposed to different social stimuli. Furthermore, our work examines relative gene expression across multiple groups—rather than just two contrasting conditions—allowing us to identify conditions with differential expression patterns (i.e. LS versus FF) and to distinguish these particular social conditions from those conditions that show more intermediate patterns.

In swordtails, stimuli that evoked the greatest differences in genomic response in females (figure 2) were attractive males (LS) and other females (FF). These two conditions also had the greatest number of candidate genes, or genes that exhibited unique expression patterns in only one out of the four conditions (77 in LS and 43 in FF), as opposed to only three in the asocial condition (figure 3). These results imply a high degree of transcriptional regulation in the brain in response to social stimuli, since different social conditions were coupled with highly specific, and often oppositional, transcriptional responses. The opposing transcription patterns also suggest that some of these genes are associated with different behaviours in opposing directions. For instance, in figure 2 we see genes that are differentially upregulated in the presence of one type of social stimulus (marked in red), are expressed at intermediate levels in the absence of social stimuli (in black), and significantly downregulated in the presence of a different social stimulus (in green). Such differential regulation that is stimulus-specific suggests that there may be conflicts, or trade-offs, between genes involved in association behaviour with the same or opposite sex.

In order to mate successfully, a female must be receptive to potential suitors. In rats, receptive females show a downregulation of certain genes associated with neural functions relative to non-receptive controls (Mong *et al.* 2003). Consistent with this finding, our study showed significant whole brain downregulation of many genes in female swordtails experiencing mate choice conditions (figures 2 and 3). For example, one of these genes, the *β*_*1*_*-adrenergic receptor*, has been shown to be critical in regulating receptivity in mammals, as it is involved in the inhibition of lordosis in rats (Etgen *et al.* 2001). Importantly, the finding that *β*_*1*_*-adrenergic receptor* expression was inversely proportional to receptivity displays in females experiencing mate choice (table 1) supports the notion that this gene plays a role in receptivity behaviour in swordtails. The downregulation of genes in the brain during mate choice, coupled with an increase in social or preference behaviour, may reflect the molecular manifestation of the transition to receptivity behaviour as well as the release of sexual inhibition. This result is consistent with the classical notion of central inhibition of behavioural output (Roeder 1935); lesions to specific CNS regions lead to a release of complex social behaviours such as aggression in insects (Huber 1955) and receptivity (lordosis) in female rats (Powers & Valenstein 1972).

Mate choice behaviour involves being discriminatory as well as being receptive. We found differential gene expression coincident with preference behaviour specific to male-only environments, while finding no such pattern in other control groups (FF or AA) or when examining other physical activities (figures 4 and 5; table 1). Specifically, of the five mate choice-associated genes examined in this regard, four (*neuroserpin* precursor, *apyrase*, *neuroligin 3* and *importin*) as well as *egr-1* covaried significantly with preference scores; with variation in preference behaviour explaining between 50 and 69% of the variation in gene expression. This is a strong indication that we captured molecular pathways associated with mate choice behaviour, although whether this represents downstream processes or some of the critical initial steps is still unknown. The significant relationship between candidate genes and preference behaviour when examining females exposed only to less attractive males is also informative (table 1; figure 5). Two of these SS females showed behavioural preference similar to the behavioural preference observed in the LS group (preference is 0 or above), and gene expression was similar for individuals showing preference regardless of male exposure group (LS and SS). This supports the idea that we captured behaviour-specific gene expression, and not expression due to variation in stimuli size.

Interestingly, the *β_1_-adrenergic receptor*, whose expression covaried inversely with receptivity, showed no significant covariation with preference. This could indicate that preference and receptivity are organized in distinct and possibly modular pathways. Pharmacological manipulations using receptor subtype-specific agonists or antagonists in combination with mate choice experiments may help uncover the specific roles of these genes in preference and receptivity.

While the important role of the *β*_*1*_*-adrenergic receptor* in female reproductive physiology has long been known, only one other gene examined in detail here has been previously linked to social behaviour: *neuroserpins* have recently been implicated in regulating mood and behaviour. Specifically, *neuroserpin*-deficient rats showed decreases in exploratory behaviour along with increases in anxiety and neophobia (Madani *et al.* 2003). Linking genes involved in the neural mechanisms of ‘exploratory behaviour’ with mate choice is an intriguing implication of our results. However, it is not yet clear that how the *neuroserpin* and *β*_*1*_*-adrenergic receptor* pathways might interact in orchestrating mate choice behaviour.

Understanding the genomic architecture of mate choice behaviour requires identifying the genes involved along with their functional interactions. Our initial examination of a subset of mate choice candidate genes and behaviour show a strong link to neuronal activity. The increased *egr-1* expression (a neuronal activity marker) observed in females showing greater preference behaviour (figure 5) supports the idea that choosing a mate is a neuronally active process. Many of the other genes showing a predictive response with preference behaviour also have an association with neurons (*importin*: involved in neuronal nuclear transport (Otis *et al.* 2006); *neuroligin*: regulating excitatory and inhibitory presynaptic contacts (Chih *et al.* 2005); and *neuroserpin*: modulates synaptic connections (Krueger *et al.* 1997)). The highly statistically significant coexpression among these genes (table 2) coupled with their neuronal association suggests possible co-regulation (as has been proposed by Berger *et al.* (1999)). This implies that a neural gene regulatory network may underlie mate preference behaviour. The lack of an association between the *β*_*1*_*-adrenergic receptor*, which modulates neurotransmitter uptake, and the other genes suggests multiple networks involved in the mate choice response.

Research in *Drosophila* has demonstrated that behaviour involves interacting gene networks dynamically responding to selection (Ranz *et al.* 2003; Anholt & Mackay 2004; Mackay *et al.* 2005; reviewed in Chenoweth & Blows 2006). A few studies have begun to identify candidate genes in *Drosophila* associated with the female response to mating (McGraw *et al.* 2004; Mack *et al.* 2006; Michalak *et al.* 2007) and courtship exposure (Lawniczak & Begun 2004) and have identified suites of olfactory-related and immune response genes as part of the genomic response. Individual variation in *Drosophila* female behavioural response coupled with candidate gene expression has not yet been shown. The variation in swordtail female response towards males of different phenotypes provides us with the opportunity to identify which genes may be critical players in the molecular cascade in the brain mediating a female choice response. By examining individual variation across females expressing mate choice behaviour, we have begun to identify particular genes that showed strong correlation with preference (*neuroserpin, neuroligin 3* and *importin*) while others show weak associations (*apyrase*) and yet another that is more predictive of other behaviours (*β*_*1*_*-adrenergic* and receptivity).

Our research provides a first glimpse at the gene networks underlying short-term responses to the social environment in vertebrates. Our next step towards understanding how these genes associated with mate choice regulate behaviour is to use detailed behavioural analysis with *in situ* hybridization of these genes to identify candidate brain areas that regulate mate preference and consequently initiate or maintain reproductive isolation between populations. Such a step will be critical to understand where in the brain the gene networks act upon. One might ask, for instance, whether mate choice discrimination is part of the dopaminergic reward circuit that has been implicated in pair-bond formation in mammals (Lim *et al.* 2004). Clearly, we are just at the beginning of a mechanistic investigation into the molecular pathways involved in mate choice, yet our results set the stage for comparing responses across closely related species that show different degrees of choice, as well as different preferences entirely, to help identify how mate choice evolves.

## Figures and Tables

**Figure 1:**
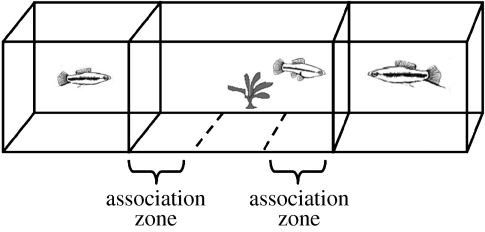
Female preference experimental tank. Stimuli (males LS or SS, females FF or nothing AA) are placed behind UV transparent plexiglas in the end zones of an experimental aquarium. Focal females are placed in the centre with three observational zones: 24 cm areas near each end zone (association zones) and a 24 cm ‘neutral’ zone in the centre. After a 5-min female acclimation period, female behaviour and association times are recorded for 15 min; stimuli are then switched and behaviour is recorded for another 15 min (to control for side biases).

**Figure 2:**
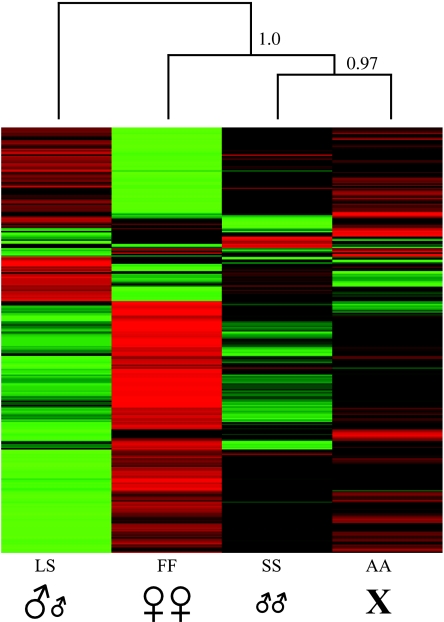
Unsupervised hierarchical bootstrapped clustering of 306 significantly (PP≥0.99) differentially expressed genes across all four experimental conditions. Green represents significantly downregulated genes, red represents significantly upregulated genes and black represents intermediate levels of expression. Bootstrapping was used to obtain confidence values for the cluster nodes. Consensus trees were constructed by repeating the hierarchical clustering 1000 times on randomly permutated expression profiles using the *hclust*, *consensus* and *heatmap* functions in R/Bioconductor. A cluster node that emerges in every single iteration will be assigned a confidence value of 1.00, whereas a node that results from only half the iterations is assigned a value of 0.50.

**Figure 3:**
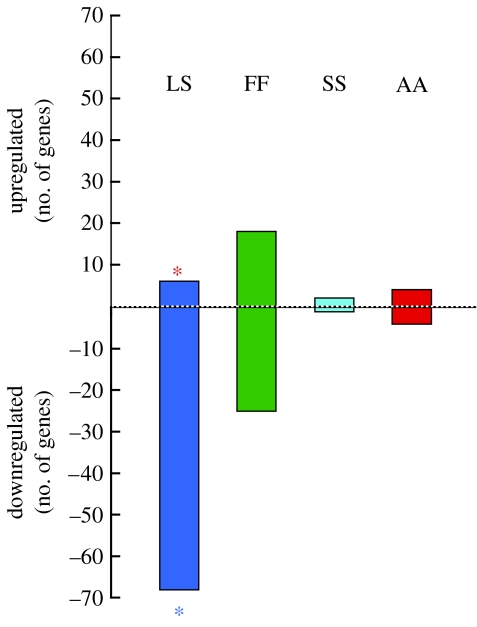
Mate choice and control-specific genes. The 128 genes (from figure 2) were significantly differentially expressed and predictive of specific social conditions (i.e. up- or downregulated in only one condition compared to all others; PP≥0.95). The *y*-axis indicates the number of unique genes significantly up- or downregulated in each environment with some genes identified to each group (complete list in table S1 in the electronic supplementary material). Red asterisk, *neuroserpin* precursor; blue asterisk, *β_1_-adrenergic receptor*.

**Figure 4:**
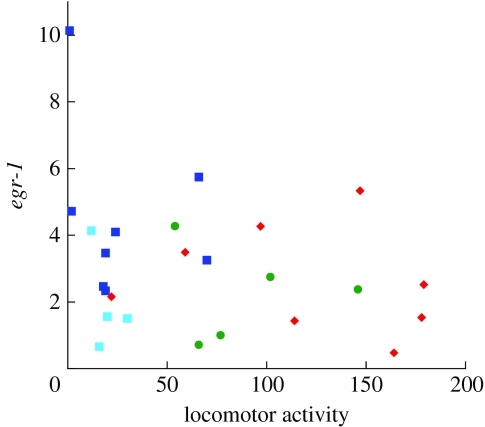
Female locomotor (non-social) behaviour and *egr-1* expression. Gene expression quantified by qPCR for 30 *X. nigrensis* females exposed to different social stimuli in experiment 2. Females exposed to the LS condition (large versus small male) are represented by dark blue squares; SS (two small males) by light blue squares, FF (two females) by green circles and AA (empty choice compartments) by red diamonds. Whole brain expression of *egr-1* (×10^8^) was normalized by *E*^avg^ ^CT^/cDNA RiboGreen. Non-social locomotor activity is measured as the total number of transits through the centre (neutral zone) of the tank during the 30-min observation period (figure 1).

**Figure 5:**
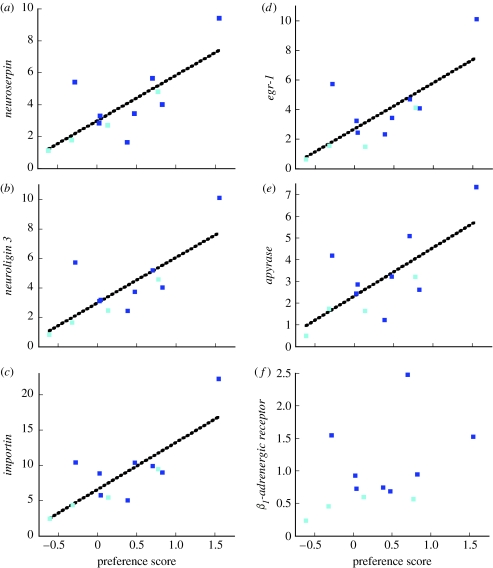
Female preference behaviour and gene expression in male exposure conditions. Gene expression quantified by qPCR for 30 *X. nigrensis* females exposed to different social stimuli in experiment 2 (LS and SS labels are the same as figure 4). Whole brain expression (normalized by *E*^avg^ ^CT^/cDNA RiboGreen) for (*a*) *neuroserpin* precursor (×10^9^), (*b*) *neuroligin 3* (×10^9^), (*c*) *importin* (×10^10^), (*d*) *egr-1* (×10^8^), (*e*) *apyrase* (×10^12^) and (*f*) *β*_*1*_*-adrenergic receptor* (×10^8^). Preference scores are defined as proportion of association time with individual 1+log(1+glide displays towards individual 1/transits through centre of tank), where association time with individual 1 is greater than individual 2. Dashed lines indicate significant correlations at *p*<0.05. There were no significant correlations between preference behaviour and gene expression in female (FF) and asocial (AA) controls (table 1).

**Table 1:** Pearson correlation coefficients, *r*, between behaviour and gene expression for females exposed to male exposure (LS and SS, *n*=12), female control (FF, *n*=5) and asocial (AA, *n*=8) conditions. (The unattractive male condition (SS, *n*=4) is reported for preference. (Significant correlations (*p*_1−tail_ values less than 0.05) are shown in italics.))

	locomotor activity	preference behaviour
*neuroserpin*		
male exposure, (SS only)	−0.22	*0.76*^a^^,^^b^ (*0.99*^a^)
FF	−0.07	0.32
AA	−0.40	0.06
*neuroligin 3*		
male exposed, (SS only)	−0.18	*0.77*^a^^,^^b^ (*0.99*^a^)
FF	0.23	0.48
AA	−0.27	−0.11
*importin*		
male exposed, (SS only)	−0.16	*0.79*^a^^,^^b^ (*0.99*)
FF	0.13	0.43
AA	−0.26	0.03
*apyrase*		
male exposed, (SS only)	−0.22	*0.72* (*0.94*)
FF	0.02	0.36
AA	−0.52	0.16
*β*_*1*_*-adrenergic receptor*^c^		
male exposed, (SS only)	−0.06	0.44 (0.80)
FF	−0.05	−0.17
AA	−0.53	0.52
*egr-1*		
male exposed, (SS only)	−0.12	*0.75*^a^^,^^b^ (*0.93*)
FF	−0.03	0.34
AA	−0.20	−0.30

a Significance remains after Bonferroni correction (where *p*_B_=0.05/12=0.004).

b Significance remains after removing the female with the highest gene expression value.

c Significant relationship between gene expression and receptivity (glide displays) during LS trials (β_1_ expression versus log-transformed glides, *r*=−0.79; *t*=3.20, *p*=0.009).

**Table 2:** Correlation matrix and (probabilities) of gene expression in male exposure groups.

	*neuroserpin*	*neuroligin*	*importin*	*apyrase*	*β_1_-adrenergic receptor*	*egr-1*
correlations						
*neuroserpin*	1					
*neuroligin*	0.987	1				
*importin*	0.943	0.969	1			
*apyrase*	0.974	0.963	0.928	1		
*β_1_-adrenergic receptor*	0.684	0.653	0.553	0.755	1	
*egr-1*	0.967	0.991	0.973	0.945	0.641	1
probabilities						
*neuroserpin*	<0.001					
*neuroligin*	<0.001	<0.001				
*importin*	<0.001	<0.001	<0.001			
*apyrase*	<0.001	<0.001	<0.001	<0.001		
*β_1_-adrenergic receptor*	0.014	0.021	0.062	0.005	<0.001	
*egr-1*	<0.001	<0.001	<0.001	<0.001	0.025	<0.001

## Supplementary Material

Table S1 Relative expression, Bayesian probabilities, and annotation of 306 differentially expressed genes from experiment 1

Table S2 Cloning, real time qPCR, and ribogreen quantification parameters for experiment 2
