# Supplementary material for: Sexual and social stimuli elicit rapid and contrasting genomic responses
Source: Proc Biol Sci. 2007 Dec 5;275(1633):393–402. doi: 10.1098/rspb.2007.1454 (PMC2212751; doi:10.1098/rspb.2007.1454)
Supplement: Supplementary file 1 [file rspb20071454s03.doc]

Table S2. Cloning, real time qPCR, and ribogreen quantification parameters

| **Cloning gene parameters. Egr-1:** Degenerate primers were designed using CODEHOP (COnsensus-DEgenerate Hybrid Oligonucleotide Primer; http://blocks.fhcrc.org/codehop.html), with consensus sequences derived from known fish and amphibian egr-1 protein sequences (*R. danio, X. laevis, A. burtoni*). The reaction parameters were 1 denaturing cycle (94° C for 2 minutes) followed by 30 amplification cycles (94° C denaturing for 30 seconds, 55° C annealing for 1:30 minutes, and 72° C elongation for 1:30 minutes) and a final 10 minute elongation cycle (72° C)  **Microarray candidate genes:** The cloning reaction parameters were 1^st^ round (Rnd 1): 1 denaturing cycle (94° C for 2 minutes) followed by 30 amplification cycles (94° C denaturing for 30 seconds, 55° C or 60° C annealing for 1minute, and 72° C elongation for 1 minute) and a final 10 minute elongation cycle (72° C). 2^nd^ round (Rnd 2) pcr parameters were 1 denaturing cycle (94° C for 2 minutes) followed by 30 amplification cycles (94° C denaturing for 30 seconds, 55° C or 60° C annealing for 30 sec, and 72° C elongation for 30 sec) and a final 10 minute elongation cycle (72° C) | | | | | |
| --- | --- | --- | --- | --- | --- |
| **gene** | **Accession number** | | **Cloning Primers** | **Size (% homology to *A. burtoni* EST)** | |
| apyrase | DQ835283 | | Rnd 1: for1 5’- GGCTACCATATAGATGGAGAACAGGC-3’  rev1 5’ – ACTTGGGAACCCAGTTCTTGTGCT-3’  Rnd 2: for1 repeated  rev2 5’- TGGTCCACTCTTTCCCGAGAC -3’ | 146 (86) | |
| β_1_  adrenergic  receptor | DQ839536 | | Rnd1: for1 5’- TCCTCGCCAGTGAACTTCCAGAAT -3’  rev1 5’- ATAACGCACAGGGTCTCGATGCT -3’  Rnd 2: rnd1 primers repeated | 367 (89) | |
| egr-1 | DQ835282 | | Rnd 1: for1 5’GACAACTACCCCAAGCTGGARGARRTNAT-3’  rev1 5’-AAGTTCCGCATGCAGATCCKRCAUTGRAA- 3’  Rnd 2: no | 1013 (84*) | |
| importin | DQ839540 | | Rnd1: for1 5’- GCTTCTGGAGACCACAGACAGG -3’  rev1 5’- GGCAGTGCTCTGAAACATCCTC -3’  Rnd 2: for2 5’- CCGATGGTCACCAGAACAACC -3’  rev1 repeated | 308 (94) | |
| neuroligin3 | DQ839541 | | Rnd 1: for1 5’- CCTTCACAATCCTCTCCACGTCT -3’  rev1 5’- ACTAAGTTCTTGCTAGGTAAAGTTCCA -3’  Rnd 2: for1 repeated  rev2 5’- AGAAAGCAAAGGCAGGATGTTCGC -3’ | 345 (82) | |
| neuroserpin precursor | DQ839542 | | Rnd1: for1 5’- TTATCGCTCTCTGTGGTCTGTGCT -3’  rev1 5’- TGGGCAAATCGGATCACATAGTGG -3’  Rnd2: for2 5’- CTGCGATGTTGATCCTGGACGTTT -3’  rev2 5’ TGTCAGGTTCTGGAGCAAGGAGAA -3’ | 379 (85) | |
| **Real time qPCR parameters:** cDNA reverse-transcribed from individual whole brain total RNA was used in a 10 μl reaction containing 1 μl cDNA template, 5 μl 2x POWER SYBR Green PCR master mix (ABI), and 5 pmol primers (Exp 1 apyrase reactions contained 2 μl cDNA template). Real time pcr parameters for exp 1 and 2 were 2 min at 50°C, 10 min at 95°C for denaturing, followed by 40 cycles at 15 sec 95° C, 30 sec 55° C, and 30 sec 72° C except for exp 2 beta1 and apyrase where amplification cycles (40) were 15 sec 95° C and 1 min 60° C. | | | | | |
| **gene** | | **Real time qPCR Primers** | | | **size** |
| apyrase | | for1 5’- GGTTATCCTGCCTGATGGAGATG -3’  rev1 5’- TCTTTCCCGAGACCACCAATG -3’ | | | 102 |
| β_1_ | | for1 5’- ACGTGTTCATCGTGTCGCTTG -3’  rev1 5’- AGAACGAGCCGTACATCTAGGAGC -3’ | | | 108 |
| egr-1 | | for1 5’- TGATTCCTGACTACCTGTTCCCC -3’  rev1 5’- GAGTAAGTGATGGCTGGTTTGACTG -3’ | | | 102 |
| importin | | for1 5’- CCGCCTACGAAGCTCTGATGG -3’  rev1 5’- GCAGCCGCTCCATGATGAC -3’ | | | 96 |
| neuroligin 3 | | for1 5’- CCAGATGACATCCCTCTGATGACC -3’  rev1 5’- GTGCTGTATGGACTCATGTTGGAG -3’ | | | 89 |
| Neuroserpin  precursor | | for1 5’- TGTCTGTTGCCCTCGGTATGG -3’  rev1 5’- GAGCAAGGAGAACTCCACACCAG -3’ | | | 113 |
| **Ribogreen quantification using total RNA and cDNA template:** Total RNA abundance was assessed using Quant-iT RiboGreen RNA reagent (Molecular Probes). Pilot experiments indicated that RiboGreen reagent measured single-stranded RNA and DNA (cDNA) with equal effectiveness (data not shown). In exp 1, we quantified template both before (DNase-treated total RNA) and after (RNase-treated cDNA) the RT reaction with an 0.96 correlation between the two measurements (data not shown). Therefore, we chose to normalize our exp 2 qPCR data to input cDNA quantification values as a better reflection of actual input target in each well. | | | | | |

*Egr-1 % homology to published *A. burtoni* Egr-1 nucleotide sequence
